# The effect of neurolinguistic programming on academic achievement, emotional intelligence, and critical thinking of EFL learners

**DOI:** 10.3389/fpsyg.2022.888797

**Published:** 2023-01-18

**Authors:** Xiuyun Zhang, Nikoo Davarpanah, Siros Izadpanah

**Affiliations:** ^1^School of Foreign Languages, Xinyang University, Xinyang, China; ^2^Department of English Language Teaching, Zanjan Branch, Islamic Azad University, Zanjan, Iran

**Keywords:** critical thinking, educational achievement, emotional intelligence, neurolinguistic programming, learning

## Abstract

Neurolinguistic programming (NLP) is a method of personal communication. This study aimed to determine the effect of NLP strategies on academic achievement, emotional intelligence, and critical thinking. Although NLP has been studied, more studies still need to be conducted on this variable contributing to language learning success. This experimental study was conducted with a pretest-posttest design with the control group in 2021. Sampling was conducted through the multistage cluster random sampling (MCRS) method, and based on the Cambridge placement test (2010), 50 students proved to be at an advanced level and participated in this study. To test the hypotheses, an ANCOVA test was employed. Participants were randomly divided into two control (25 people) and experimental groups (25 people). They were performed on the experimental group during 12 sessions of 90 min of the strategic training in NLP. In the experimental group, the mean and std of critical thinking was 16.24 ± 2.59 in the pretest, which increased to 18.88 ± 2.77 in the posttest; the mean and std of academic achievement was 155.02 ± 15.90 in the pretest, which rose to 171.70 ± 10.83 in the posttest and the mean and std of emotional intelligence was 96.51 ± 12.44 in the pretest, which increased to 118.28 ± 6.18 in the posttest. The results of data analysis by covariance method showed that NLP was practical on learners' academic achievement, emotional intelligence, and critical thinking. Justifications and implications for the study's findings and suggestions for further research are presented.

## Introduction

It is becoming increasingly difficult to ignore neurolinguistic programming (NLP). The most crucial role of NLP is to help humans better communicate with themselves and control negative emotions and anxiety (Nompo et al., 2021). NLP is considered an assistive technology that can help teachers and learners develop outstanding skills such as critical thinking, academic achievement, emotional intelligence, self-efficacy, and empathy (Begum et al., 2022). These skills are necessary for success and achievement in English teaching education and to help learners achieve excellent results (Anjomshoaa et al., 2021). NLP methods and tools can be used in the classroom to create impressions about learner relationships, actions, learning and performance, and teaching efficiency (Keezhatta and Omar, 2019). NLP also helps language teachers strengthen the educational environment and carry out supportive and effective interactions, thereby improving academic performance (Anjomshoaa et al., 2021). Facilitation of the learning process is another factor of the NLP technique in education (Nazim and Yousaf, 2021). The characteristics of an individual's motivation should be considered to have effective interaction with a learner (Espinales and Moreno, 2021). To increase motivation and the knowledge aptitudes of individuals, in this respect, multiple intelligences contribute to numerous cognitive and learning styles, and effective and specific thinking strategies (El-Ashry, 2021).

One of the most important goals of education in the 21st century is how to educate learners who are prepared to face the changing society and complexities of the information age. Accordingly, the promotion of thinking in schools and educational centers is of great importance, and this is not only achieved through the transmission of information to the minds of students but also requires a fundamental change in curricula and teachers' attitudes toward their duties (Gehlbach and Robinson, 2021). Teaching and change are in a direction that leads to strengthening the student's thinking power.

Emotional intelligence (EI) was first coined in the 1990s by two psychologists, Mayer and Salovey (1997). According to Oxford Dictionary, EI is the ability to understand emotions and behave appropriately in different situations. As stated by Mayer and Salovey (1997), EI is the ability to monitor one's own and others' emotions, discriminate among them, and to guide one's thinking and actions.

The academic achievement term is how students achieve pre-determined educational goals and expect to achieve them in their learning endeavors. Ramos et al. (2021) believe that highly motivated learners succeed in homework that motivated learners to persevere longer in completing tasks than less motivated learners, rather than failing on their own. They do not attribute it to external factors such as difficulty or chance or redouble their efforts to succeed.

Academic achievement, EI, and critical thinking are essential components of NLP and play a vital role in learning (Sunitha et al., 2021). Recent developments in NLP have highlighted the need for it. In recent years, there has been an increasing interest in teaching strategies of NLP. However, a significant problem with NLP methods is finding appropriate ways. Although NLP as an essential component of language acquisition facilitates learning, still more studies need to be conducted on these variables that contribute to success in education. This research aims to study the effect of NLP on academic achievement, EI, and critical thinking of EFL advanced learners.

## Research questions

This study seeks to address the following questions:

1. Do NLP Strategies Play an Influential Role in Learners' Critical Thinking in Advanced Language proficiency?
2. Do NLP strategies influence/contribute to learners' academic achievement in advanced language proficiency?
3. Do NLP strategies influence the learners' EI in advanced language proficiency?

## Literature review

### Neurolinguistic programming

Neurolinguistic programming may show human interaction and communication that encourages examination and generation prevalence in clinical and nonclinical settings (Wake and Leighton, 2014). It emphasizes the mental involvement of people and the constructive reality. It is an internal representation of the experience and how people relate to themselves and others. It mainly depends on non-verbal communication (Anderson, 1986; Tosey et al., 2005). NLP was set up in the 1970s by Bandler, a mathematician and data researcher, and Grinder, a language specialist. Bandler and Grinder created a modern technique to recognize and code viable practices from a run of professionals and theories, their models, and techniques and making them transferable to other individuals in an endeavor to take after their case and accomplish the ideal performance (Tosey and Mathison, 2003; Tosey et al., 2005). NLP professionals center not on hypotheses but on the words and how they utilize them, their tone of voice, the rhythm of their discourse, their signals and developments, and their breathing patterns (Anderson, 1986).

The substantial resource of NLP was the Potential Human Movement, of which Maslow (1943) and Rogers (1971) were the leading pioneers. Koerzybski, Virginia Satir, Fritz Perls, Bandura, Erickson, and Bateson have had a considerable influence on NLP and are considered by many as the originators of NLP. Particularly, NLP professionals see that all people can completely utilize the resources for their full advancement. Individuals are obligated to manage their cognitive maps by altering language forms, considerations, sentiments, experiences, and physiology. NLP may be a solution-based approach, as it does not search for the roots of problematic behaviors but looks to persuade behavior to alter. NLP tackles qualities centered on implications, conceivable outcomes, and arrange. It almost talks about criticism, not “failure,” which opens up what works, proposing that it is best to undertake something distinctive that does not work (Linder-Pelz and Hall, 2007).

Moreover, NLP specialists recognize the significance of language in making convictions and thought designs that contribute to a more fantastic picture of who we are. The role of NLP is to assist people in adjusting to the improper nature of these negative convictions and contemplations (Kudliskis and Burden, 2009). NLP has since accomplished ubiquity as a strategy for communication and individual advancement. It is now recognized as a compelling model of psychotherapy within the United Kingdom. NLP is utilized by proficient professionals of numerous sorts, including teachers, directors, coaches, sales representatives, showcase analysts, counselors, specialists, doctors, and legal counselors (Tosey et al., 2005). However, it has been the subject of significant feedback in academic and clinical brain research divisions due to the need for vigorous clinical proof (Witkowski, 2010; Wake and Leighton, 2014).

### NLP and metacognition

Metacognition alludes to consciousness-raising aptitudes and procedures through which people coordinate their activities toward greatness. Metacognition includes individuals' capacity to watch, direct, and adjust their inner cognitive forms, recognize the distinction between functional and dysfunctional states of intellect, and deliberately select those states that stir the complete extent of their capacities and personality (Drigas and Mitsea, 2020). Drigas and Mitsea (2021) depicted metacognition employing a layered structure. Each layer speaks to a particular state of intellect where an ever-higher control framework reacts to the need to make more theoretical mental representations, upper-class inspirations, convictions, and feelings. The ideal state of metacognition coincides with the state of mindfulness (Drigas and Mitsea, 2020). NLP recognizes individuals' capacity to require control of themselves to open their authentic potential. NLP analyzes how the cognizant intellect works by centering on the subconscious factors (i.e., convictions, values, demeanors, and recollections) that certainly impact conscious processes (Furduescu, 2019).

### NLP and positive psychology

Positive psychology research is the logical consideration of the conditions and the forms that contribute to the ideal working of individuals and groups. In line with NLP, positive psychology underpins the thought that convictions shape our feelings and activities. Research has uncovered that individuals are hardwired to pessimism, and as a result, they may be disrupting themselves. Positive psychology looks for the variables and how individuals feel delighted, constructs strengths, versatility, and creates the complete range of human experience. Due to this, positive psychology analysts suggest a few preparing techniques that develop positive convictions and a sense of individual control (Gable and Haidt, 2005). There is considerable proof from well-controlled thinks that abilities expanding flexibility, appreciation, positive considering, positive feelings, engagement, and meaning can be viably instructed to schoolchildren. Schools may be an excellent area for wellbeing activities. Positive instruction avoids side effects of misery and uneasiness, diminishes behavioral issues, upgrades social aptitudes, and brings hope. Moreover, positive thinking preparation is synergistic with better learning, improved consideration forms, and more inventive and all-encompassing thinking (Seligman et al., 2009).

Neurolinguistic programming is a field of psychology that deals with internal processes (Gehlbach and Robinson, 2021). Grinder and Bandler developed a new collaborative communication method for language teaching in the early 1970s (Purwanto et al., 2022). Neuro, which relates to the nervous system; linguistic, which are words, pictures, and sounds; and programming, which relates to the program to run our thoughts are three elements of NLP (Gran, 2021). Some NLP strategies, systems, and designs are used for efficient interaction and individual modification. According to Grinder and Bandler, neural processes, language, and social patterns are interrelated, and individuals can modify them to access the anticipated objectives.

From an NLP point of view, individuals are considered interactive collaborators with stable, patterned associates between neurons, linguistics, and programming (Gran, 2021). Identifying people's thoughts as an individual who has the desired sense of taking in information that should be revealed to help learners increase their learning is one of the vital abilities of NLP. Modeling is the straightforward principle of NLP in which recognizing the particular series of thoughts in an individual and instructing that construction to a new individual (Jahan et al., 2022).

#### Academic achievement

One of the most important issues in educational systems is to create the necessary conditions for academic success. Many research findings have also been confirmed by the effects of nervous planning teachers, the motivation of progress and academic achievement. In this regard, Schunk (1995); Zimmerman and Bandura (1994) showed that targeting in improving educational and academic performance has an influential role. In a general view, the factors affecting academic achievement can be divided into two categories, namely, external and internal factors. External factors include the learning situation, learners' participation in learning, textbooks, teaching aids, and teaching methods. According to the researchers, motivated learners succeed more than demotivated learners in their homework (Hübner et al., 2022).

In this respect, this question arises about improving advanced learners' achievement motivation and fortifying their EI based on NLP. Most studies in NLP have only focused on NLP techniques and other aspects and components of the teaching-learning process. Researchers have not treated NLP strategies on learners' academic achievement motivation and EI in advanced language proficiency. The previous studies illustrated that secondary school levels had changed significantly in favor of NLP (Low et al., 2020). One of the most important goals of education in the 21st century is educating learners to have the necessary readiness to confront society and the complexity of the age of information. Therefore, the promotion of thought and thinking in schools and educational centers is of great importance, and this is not just about the transfer of information in the minds of students but also requires a fundamental change in curriculum and change in the attitude of learning toward their duties to strengthen their thinking (Singh, 2011).

#### Emotional intelligence and NLP

According to Oxford Dictionary, EI is “the ability to understand your emotions and other people's emotions and behave appropriately in different situations.” Mayer and Salovey (1993, p. 433) stated that EI is “the ability to monitor one's own and others' emotions, discriminate among them, and use the information to guide one's thinking and actions.” Different abilities of EI can be enhanced by teaching (Mayer and Salovey, 1997). People have other talents to recognize, process efficiently, and control their feelings (Gardner and Stough, 2002). Five scopes of EI, such as self-awareness, self-regulation, self-motivation, empathy, and social ability, were categorized (Resmisari and Sitepu, 2022). Based on the NLP methods, people can handle emotional challenges when coping with difficulties (Weare and Gray, 2003; Saffaryazdi et al., 2022). Various ways have been considered in NLP to increase each dimension of EI, such as for self-awareness (values hierarchy and goal setting), self-regulation (dissociative technique), self-motivation (associative approach), empathy (matching and mirroring), for social skills (rapport).

There has been an increasing amount of literature on NLP strategies in teaching and learning environments. Likewise, an extensive growing literature has investigated NLP techniques in education and EI. It has been demonstrated that NLP has an operational instrument for increasing teacher-learner rapport and stimulating collaborative learning situations (Keezhatta and Omar, 2019). NLP techniques have been identified as major contributing factors to increasing EI and teaching-learning success. However, one question that needs to be asked is whether NLP strategies practically contribute to the learners' academic achievement, critical thinking, and EI among advanced learners.

Despite the importance of EI, academic achievement, and critical thinking, educators realize their importance, and their use in educational programs has been prolonged. Learners abundantly acknowledge the inadequacy of their readiness for such interventions and their reluctance to take on such influential roles. It is often thought that our teachers have EI, academic achievement—critical thinking, and their skills and can develop these skills in learners, so not much effort has been made in this area. Therefore, this study examines whether such training can effectively increase learners' EI-academic achievement and critical thinking by developing a training program for NLP strategies. It is proposed that methods such as confirmation, visualization, securing, reframing, roleplaying, and part modeling may have a positive effect on the brain coming about in learning advancements and behavioral adjustment (Tosey and Mathison, 2003; Kudliskis and Burden, 2009; Lashkarian and Sayadian, 2015).

### Critical thinking and NLP

In critical thinking, they should adapt themselves to the continuous changes of their environment and internalize the flow of information, while they also reflect on both the personal and professional levels (Biggs, 2003; Chang et al., 2022; Khurana and Lee, 2022; Purwanto et al., 2022). Currently, the tendency to teach thinking in the institute has become more general. According to Paul's viewpoint (Paul, 1997), the learners should have critical thinking characteristics, such as mental independence, mental curiosity, commitment to reasons, open mind, straightforward intellectual, and courageousness. Critical thinking is considered an essential and unavoidable component and the personality trait of educated people. According to Walsh and Paul (1988) and Vieira and Gaspar (2013), critical thinking is a skill that all individuals can gain. Critical thinking is not originally associated with the growth of individuals but should be trained. There is less agreement on the concept of critical thinking. Dewey and Zugsmith (1933) and Yeh (2004) consider a philosophical view of critical thinking, including the exploratory, distinction, and examination of various options about a problem. Paul (1997) poses crucial thinking in two meanings: in its limited sense, critical thinking is a collection of technical skills used only to criticize opposite views. In the broad sense, critical thinking is the study of self-regulatory tendencies and tendencies in the heart of hidden beliefs, including the preferences and characteristics of personality, attitude, and nature. In recent decades, psychologists and researchers have attempted to improve individual capabilities by providing and developing strategic intervention programs. In California in the early seventies, two professors, Payler and Dr. Grianman, founded NLP at the University of Santacruz to provide successful psychotherapy patterns (Breton, 1964).

## Method

In the first training session of NLP Strategies, along with the introduction, the definition of NLP, and its application, subjects in both groups were presented with the Schering Emotional Intelligence (EQ), Academic Achievement (AA), and Critical Thinking Questionnaires (CCTSTS) to complete. Then, the training program of NLP strategies was presented to the experimental group for 12 sessions. While summarizing and reviewing the material in the final session, both groups were given the Scherering Emotional Intelligence, Academic Achievement, and Critical Thinking Questionnaires of California to answer again.

### Design of study

This research is an experimental type and a pretest, and posttest design with a control group. This study was quantitative, as the research analysis used categorical and nonparametric data to test the hypotheses.

#### Participants

The participants of this study were 50 advanced female learners in Zanjan. Zanjan Province has eight cities; among these cities, Zanjan city was chosen. Zanjan city consists of 110 institutes, and two institutes were randomly selected. In 2021, there were 110 institutes, and among them, two institutes were randomly chosen for the study. Each of them has 16 classes. Two of them were selected, and in each class, there were 25 students. The advanced level of learners of 2 institutes, Jahad Daneshghahi and Safir, were randomly chosen. There were eight advanced classes, and two were randomly selected for this study.

### Sample size

The sample size is calculated using the following equation:


$$\begin{array}{l} n\;=\;\frac{{\left(z_{1\;-\;\frac{\alpha }{2}}\;+\;z_{1\;-\;\beta }\right)}^{2}\left(S_{1}^{2}\;+\;S_{2}^{2}\right)}{{\left(\mu_{2}\;-\;\mu_{1}\right)}^{2}} \end{array}$$

Here, assuming z_(1−α/(2))_ = 1.96 and z_(1−β)_ = 0.8 and considering the values of $S_{1}^{2}=$ 6.4, $S_{2}^{2}=$ 6.767 and μ_2_ = 18.100, μ_1_ = 16.100, the sample size for each group 25.

### Instruments

The following instruments were employed to collect the required data for this study.

### Cambridge Placement Test (2010) by Cambridge University Press

#### California critical thinking skills test

This variable is obtained from 34 questions from the Facionc and Facione (1992) Questionnaire. In this questionnaire, each subject has four options, and as there is only one correct answer, the subject gets a score of 0 or 1 in each article. The overall score of the person is between 0 and 34. This questionnaire includes the components of evaluation (13 items), inference (11 items), and analysis (9 items), as well as deductive reasoning (14 questions) and inductive reasoning (15 questions). Khalili and Hossein (2003) has examined the validity and reliability of the test. Thus, the reliability of the test was obtained by the Koder-Richardson method of 0.62, and the result of factor analysis in determining the validity of the structure indicated that the test consists of five factors (analysis, inference, evaluation, deductive reasoning, and inductive reasoning). All five factors had a positive and high correlation with the total test score. The test also showed critical thinking skills between nursing and philosophy students. Facione (1990; 1992) reported the reliability of the test by Richardson's Koder method as 0.7–0.68. Eslami (2003) re-evaluated the critical thinking of California's crucial thinking on student-teachers in Tehran Teacher Training Centers and reported a reliability of 0.73.

#### Sherer emotional intelligence questionnaire (EQ)

This test contains 33 items that are graded on a 5-point Likert scale. This questionnaire includes self-motivation subscales (questions 31, 27, 26, 24, 21, 20, 15, 9, 1), self-awareness (questions 6, 10, 12, 14, 32, 33), self-control (questions 2, 5, 11, 16, 18, 23, 30), empathy (questions 3, 4, 17, 22, 25, 29), and social skills (questions 7, 8, 13, 19, 28). The lowest score obtained in this test is 33, and the highest is 165. A high score on this test indicates greater emotional intelligence. Mansouri (2001) reported Cronbach's 33-item test at about 0.84. He also obtained the construct validity of this test by measuring the correlation of subjects' scores in this test and the Cooper-Smith self-esteem test (*r* = 0.63). Bankdari (2005) evaluated the validity of the structure, the Shrink test with Meyer's emotional intelligence test, and the correlation coefficient of these two tests reported (*r* = 0.62). In this study, the reliability coefficient was calculated by the internal consistency of items, and the amount of Cronbach's alpha was 0.74. The construct validity of this test was examined by correlating the scores of this test with the Cooper-Smith self-esteem test on a sample of 30 people; the findings indicate that the correlation between the scores of these two tests is statistically significant (R= 0.63, *p* = 0.01), and it can be said that the emotional intelligence test has sufficient construct validity. To evaluate the reliability of this test, the internal consistency of the test, which was performed on a sample of 400 people, by Cronbach's alpha method of 0.84 for the whole test, it can be said that the Shrink emotional intelligence test (a standardized form of 33 questions) has a good validity (Mansouri, 2001).

#### Academic achievement test

This questionnaire consists of 48 questions from the standard questionnaire by Pham and Taylor (1999) classified as a 5-point Likert scale from very high with code 5 to none with code 0. The highest score is 240, and the lowest score is 48. Khaledian et al. (2013) have confirmed its validity through content validity by experts and specialists. Khaledian et al. (2013) obtained the reliability of the questionnaire using Cronbach's alpha coefficient of 0.82.

Regarding the reliability of the questionnaires, the Cronbach's alpha index for the critical thinking questionnaires was 0.805, academic achievement was 0.780, and emotional intelligence was 0.847, and the questionnaires have the necessary reliability.

### Procedure

#### Intervention method

The training program of NLP strategies was organized in 12 sections and was implemented in 12 sessions of 90 min. The educational package in this study is taken from the book NLP written by Reddy and Burton and translated by Ghahremani.

**Session 1: (Familiarity)** Familiarity with the subjects, the definition of NLP and its application, conducting a pretest.

**Session 2: (Pattern discovery and modeling)** Discovering the communication pattern in NLP, taking full responsibility for behaviors and reactions, understanding how others communicate with each other, communicating effectively, letting go of feelings and emotions, focusing on results, and performing class exercises.

**Session 3: (Understanding the subconscious mind)** Recognizing the difference between the conscious and the subconscious mind, understanding how the brain works, overcoming fears (managing phobias), beliefs, and values, and doing class practice.

**Session 4: (Life management, taking control of life)** Taking control of memories, the path to excellence, the four-part formula of success, turning the wheel of life, goal book, doing class practice.

**Session 5: (Sensory awareness)** Recognition and control of the five senses, listening to the world of words, understanding the importance of the eyes, three dimensions of hearing, sight, and emotion, doing class practice.

**Session 6: (Creating intimacy)** Knowing why intimacy is important, dealing with difficult people, the ability to say no, raising your choices to respond and react, understanding the experiences of others, and doing classroom exercises.

**Session 7: (Mental plans)** Take a look at mental plans and time patterns, discover mental plans and behaviors, understand similarities and differences, time perspective, combine mental techniques, and do class exercises.

**Session 8: (Experience control)** Setting and controlling sensory inputs, letting go of limiting beliefs and creating invigorating beliefs, whistling technique, understanding very important senses, and doing class practice.

**Session 9: (Working with logical levels)** Understanding logical levels, using the NLP model to achieve coordination, discovering your goals, focusing on work and life, forming work teams, and practicing logical levels.

**Session 10: (Time management)** Discover the timeline, understand the timeline, let go of the negative effects of inappropriate emotions and feelings, change the language script, organize time, create the future in the timeline, and do class exercises.

**Session 11: (Going to the heart of the matter: the meta pattern; discovering the language of ecstasy: the Milton model)** Recognizing the meta pattern, gathering specific information with the meta pattern, applying the meta pattern, influencing the listeners, using the Milton model, conducting class training.

**Session 12: (Summary)** Summarize and review the material with subjects' help and perform posttest.

## Results

### Descriptive findings

In this section, researchers first examined the central indicators (mean) and dispersion (standard deviation) of research variables.

The results in Table 1 showed that the mean pretest in the critical thinking variable in the experimental group was 16.24 ± 2.59 and in the control group equaled 15.80 ± 2.45. The mean posttest in the critical thinking variable in the experimental group was 18.77 ± 2.77, and in the control group was 16.52 ± 2.20, which shows that the critical thinking score in the experimental group increased after the intervention.

**Table 1:** Central indices and posttest dispersion in control and experimental groups.

		**Experimental group**				**Control group**			
		**Mean**	**Std. deviation**	**Minimum**	**Maximum**	**Mean**	**Std. deviation**	**Minimum**	**Maximum**
Pretest	Critical thinking	16.24	2.59	11.00	22.00	15.80	2.45	12.00	20.00
	Academic achievement	155.02	15.90	129.00	181.00	156.19	15.85	129.00	181.00
	Emotional intelligence	96.51	12.44	71.00	113.00	98.56	14.03	70.00	133.00
Poos-test	Critical thinking	18.88	2.77	14.00	25.00	16.52	2.20	12.00	21.00
	Academic achievement	171.70	10.83	156.00	193.00	157.96	15.55	131.00	183.00
	Emotional intelligence	118.28	6.18	105.00	132.00	100.16	13.16	70.00	133.00

The mean of the pretest in the academic achievement variable in the experimental group was 155.02 ± 15.90, and in the control group was 156.19 ± 15.85. The mean posttest in the academic achievement variable in the experimental group was 171.70 ± 10.83, and in the control group was 157.96 ± 15.55, which showed the scores of academic achievement in the experimental group increased after the intervention.

The mean of the pretest in the EI variable is 96.51 ± 12.44 in the experimental group and 98.56 ± 14.03 in the control group, and the mean of posttest in the EI variable in the experimental group is equal to 118.28 ± 6.18 and in the control group is equal to 100.16 ± 13.16 and shows that the score of EI in the experimental group has increased after the intervention.

### Inferential analysis

Analysis of covariance was used to test the research hypotheses. Covariance analysis is a comprehensive type of analysis of variance. While comparing the means of one or more groups and estimating one or more independent variables, the effect of intervening variables, or covariates, is excluded from the equation process:

### Assumptions of analysis of covariance

Before analyzing the research data, the assumptions of the ANCOVA test, i.e., data normality, variance homogeneity, regression homogeneity, and linearity of the scattering variable and the independent variable, are examined. The results are presented in Tables 2, 3.

**Table 2:** Data normality test and variance homogeneity.

		**One-sample Kolmogorov-Smirnov test**				**Test of homogeneity of variances**			
		**Experimental group**		**Control group**					
**Variables**		**Test statistic**	***P*-value**	**Test statistic**	***P*-value**	**Levene statistic**	**df1**	**df2**	***P*-value**
Critical thinking	Pre-test	0.097	0.200	0.169	0.064	0.067	1	48	0.797
	Post-test	0.164	0.095	0.131	0.200	0.005	1	48	0.944
Academic achievement	Pre-test	0.105	0.200	0.126	0.200	0.084	1	48	0.773
	Post-test	0.154	0.130	0.119	0.200	2.506	1	48	0.120
Emotional intelligence	Pre-test	0.097	0.200	0.112	0.200	3.085	1	48	0.085
	Post-test	0.126	0.200	0.147	0.168	3.883	1	48	0.072

**Table 3:** Reception of homogeneous regression slope.

	**Reception of homogeneous regression slope**			**Correlation pretest and posttest**		
	**Variable**	**F**	***P*-value**	**variable**	**F**	***P*-value**
Hypothesis 1	Critical thinking group	1.161	0.251	Critical thinking	94.354	0.000
Hypothesis 2	Academic achievement group	1.508	0.162	Academic achievement	152.159	0.000
Hypothesis 3	Emotional intelligence group	0.937	0.423	Emotional intelligence	64.203	0.000

**Default 1—Pretest:** This default is observed, and before implementing the independent variable, i.e., NLP, covariate (pretest) has been done.

**Defaults 2 and 3—Normality and homogeneity of variables:** The default normality of data was evaluated by the Kolmogorov-Smirnov test and homogeneity test of variance with Leven's test, the results of which are presented in Tables 2, 3.

According to the results of the Kolmogorov-Smirnov test in Table 2, the hypothesis of normality of research variables by control and experimental groups was confirmed (*P*-value > 0.05). Also, according to Table 2, the Leven test accepted the hypothesis of homogeneity of variances (*P*-value > 0.05).

**Assumptions 4 and 5 regression slope homogeneity and confirmation of the auxiliary variable effect hypothesis:** The results of this were obtained through analysis of covariance, which is presented in Table 4.

**Table 4:** Results of analysis of covariance for critical thinking.

		**Mean**		**Analysis covariance**					
**Variable**		**Experimental**	**Control**	**Type III sum of squares**	**df**	**Mean square**	**F**	***P*-value**	**Partial eta squared**
Critical thinking	Pretest	16.24	15.80	49.740	1	49.740	23.368	0.000	0.332
	Post-test	18.88	16.52						

According to the results in Table 3, the regression slope homogeneity hypothesis was accepted through analysis of covariance (*P*-value > 0.05).

According to the results in Table 3, the choice of auxiliary variable (pretest) as a covariate is confirmed in this study (*P*-value < 0.05).

### Investigating research hypotheses

#### The first hypothesis of research: NLP significantly affects critical thinking

Covariance analysis was used to test the hypothesis. As seen, the presuppositions for analysis of covariance are examined, and these defaults are established. The result of covariance analysis is recorded in Tables 4, 5.

**Table 5:** Results of analysis of covariance for academic achievement.

		**Mean**		**Analysis covariance**					
**Variable**		**Experimental**	**Control**	**Type III sum of squares**	**df**	**Mean square**	**F**	***P*-value**	**Partial eta squared**
Academic achievement	Pretest	155.02	156.19	2,665.169	1	2,665.169	61.604	0.000	0.567
	Post-test	171.70	157.96						

As noted in Table 4, NLP significantly affects critical thinking (*P*-value = 0.001, *F* = 23/368). Therefore, the mean of the two groups was significantly different in the posttest after adjusting the pretest scores. As seen in the tables, the mean of critical thinking scores in the control group in the pretest was 15/80 and in the posttest was 16/52, while the mean of variable in the experimental group in the pretest was 16/16, and in the posttest was 18/88. Due to the significant difference between the scores in the posttest in the control and experimental groups, it was concluded that by removing the pretest factor (covariate), the NLP approach increases the scores of critical thinking. The eta power potential is 33% of the critical thinking variability in the experimental group derived from NLP.

#### The second hypothesis of the research: NLP significantly affects academic achievement

Analysis of covariance was used to test the above hypothesis. The necessary assumptions for the covariance study have been examined, and these assumptions are valid. The results of the study of covariance are recorded in Table 5.

As seen in Table 5, NLP on academic achievement has a significant effect (*P*-value = 0.001, *F* = 61.604). Therefore, it was concluded that the mean of the two groups in the posttest after adjusting the pretest scores (covariate) was significantly different from each other.

As can be seen in the tables, the average academic achievement score in the control group in the pretest was 156.19 and in the posttest was 157.96, while the mean of this variable in the experimental group was 155.02 in the pretest and 171.70 in the posttest. Due to the significant difference between the scores in the posttest in the control and experimental groups, it was concluded that by removing the pretest factor (covariate), the NLP approach increases academic achievement scores. According to the size of the effect coefficient, the second power of eta is 57% of the variability of academic achievement in the experimental group resulting from NLP.

#### The third research hypothesis: NLP has a significant effect on emotional intelligence

Analysis of covariance was used to test the above hypothesis. As observed, the necessary assumptions for the analysis of covariance have been examined, and these assumptions are valid. The results of the study of covariance are recorded in Table 6.

**Table 6:** Covariance analysis for emotional intelligence.

		**Mean**		**Analysis covariance**					
**Variable**		**Experimental**	**Control**	**Type III sum of squares**	**df**	**Mean square**	**F**	***P*-value**	**Partial eta squared**
Emotional intelligence	Pretest	96.51	98.56	4,641.509	1	4,641.509	101.756	0.000	0.684
	Post-test	118.28	100.16						

As seen in Table 6, NLP has a significant effect on EI (*P*-value = 0.001, *F* = 101/756). Therefore, it was concluded that the mean of the two groups in the posttest after adjusting the pretest scores (covariate) was significantly different from each other. As seen in the tables, the mean of EI scores in the control group in the pretest was 98.56, and the posttest was 100/16, while the mean of this variable in the experimental group in the pretest was 96.51 and in the posttest was 118.28. Due to the significant difference between the scores in the posttest in the control and experimental groups, it was concluded that by removing the pretest factor (covariate), the approach of NLP increases the scores of EI. According to the size of the effect coefficient, eta's second potential is 68% of the EI variability in the experimental group derived from NLP.

## Discussion

Analysis of the results obtained using the covariance statistical method showed that NLP increased the critical thinking, academic achievement, and EI of EFL learners. This research seeks to address the following hypotheses:

1. NLP strategies play an influential role in learners' critical thinking in advanced language proficiency.
2. NLP strategies influence/contribute to learners' academic achievement in advanced language proficiency.
3. NLP strategies influence the learners' EI in advanced language proficiency.

Regarding the first hypothesis, analysis of the results obtained using the statistical method of covariance showed that the strategic training of NLP increased learners' critical thinking. The obtained results are consistent with the results (Biggs, 2003; Vieira and Gaspar, 2013; Chang et al., 2022; Khurana and Lee, 2022; Purwanto et al., 2022). Critical thinking to learners is considered a new standard of learning skills. Learners with such skills tend to use student-centered learning approaches. Such learners are analytical and rational about their learning and use the feedback provided by the learning process. The tendency to think critically is related to the learning of thinkers and thus contributes to the professional development of learners. Learners with a critical thinking attitude and disposition are aware of their behavior and have a mental openness to learning and acting logically (Yeh, 2004).

As mentioned earlier, it can be said that in the NLP training process, learners were taught strategies such as understanding the correct use of logical levels, question-and-answer techniques, using the Meta-model and Milton model, and taking these strategies into account. These various changes were made in the learners so that it can be said that the logical levels in the NLP enable the individual to think in a situation or experience about the components of that situation or experience. It enables logical levels to understand what is happening in the world around them; thus, being aware of the structure, pattern, content, events, relationships, and components around them, they will be aware when they are faced with a dilemma, and they can use this strategy to reach the right decision. It can also be said that another strategy of NLP, namely the meta-model, is a device that allows a person to have more complete and better access to the experiences of others. The meta-pattern poses questions that familiarize the individual with the omission, generalization, and distortion of others. Meta-model questions provide powerful oral tools in business, coaching, education, treatment, and life. These tools allow the individual to use language to gain a clear understanding and get closer to the experience of others. Milton's NLP model enables individuals to access their subconscious resources and make the necessary changes to solve their problems. Humans have an extraordinary ability to interpret the words of others differently, even if they are completely meaningless. Sometimes what we say has no special meaning and we give others the opportunity to give our words whatever meaning they like. When language structure is deliberately blurred, different people can get what they need from what we have to say in a way that benefits them. In this model, the goal is to reduce the details of using ambiguous language. In this model, the goal is to reduce the details and use ambiguous language. NLP shows how powerful and effective language is. For this reason, by choosing the right language and more awareness, one can control the reactions of others and himself. Therefore, it can be argued that teaching NLP strategies have increased learners' critical thinking.

Regarding the second hypothesis, the findings of this study indicate that NLP had a significant effect on the academic achievement of the experimental group compared with the control group, and these findings are consistent with the results of other studies (Zimmerman and Bandura, 1994; Schunk, 1995; Anjomshoaa et al., 2021; Espinales and Moreno, 2021; Ramos et al., 2021). Because in similar research, researchers emphasize the important role of goal setting and time management in learners' academic achievement. Singh (2011), Low et al. (2020), and Hübner et al. (2022) found that the knowledge of highly advanced learners is often self-regulated; in other words, these learners set their learning goals more precisely than those with low-progress learners and evaluate their progress toward the goal more systematically. Skills training is the expression of a constructive intervention method to improve interpersonal relationships and dialogue with others. This affects learning because excellent interpersonal relationships lead to confidence-empathy and an increased sense of belonging-self-esteem and create a positive atmosphere for learning.

Regarding the effect of goal setting on learners' academic achievement, researchers have found that choosing goals that are too easy or too difficult causes people to lose their motivation to succeed. In contrast, when people choose goals with medium and appropriate difficulty, their motivation to progress increases. Considering this issue, goal setting in the field of NLP by using the smart model (i.e., specific goals—measurable—achievable—real and timely) can increase their motivation to succeed. Changes in learners' motivation for progress can be due to time management training that has manifested itself in self-control. If time management is focused on specific goals such as education, one can expect to increase the commitment and responsibility of the individual in this area. With increasing personal commitment, goal-focused activities increase and as a result lead to success in achieving goals, which in turn increases the motivation for personal progress (Ramos et al., 2021). As noted in the personality traits of highly motivated individuals, these individuals are more resilient to social pressures and are more likely to adapt to others. With regard to this feature, in the shadow of NLP training and by training the skills of expressing social interactions, it increases and strengthens the inner aspects of the person in the face of life events, and this in turn increases the motivation for progress.

Regarding the third hypothesis (emotional hypothesis), analysis of the results obtained using the statistical method of covariance showed that the strategic training of NLP increased learners' EI. The result is consistent with the research results (Gardner and Stough, 2002; Keezhatta and Omar, 2019; Drigas and Mitsea, 2020, 2021; Resmisari and Sitepu, 2022; Saffaryazdi et al., 2022).

In explaining the mentioned result, it can be stated that in teaching NLP, various strategies such as pattern discovery and modeling, subconscious mind perception, life management, sensory awareness, intimacy, and time management were taught to teachers. Research has shown that teaching emotional and social skills as a model of intervention, which is called strengthening EI, has a vital role in improving the quality of interpersonal, social relationships, and promoting mental health and contributes to the balance of work and life) Gardner and Stough, 2002).

According to the NLP communication model, when people behave in a certain way (external behaviors), a particular reaction is evoked in us (internal reaction). This reaction causes us to behave in a certain way (our external behavior), and in this way, an internal reaction will be created in the other person (internal reaction) and thus this cycle will continue (Mayer and Salovey, 1997). NLP provides the individual with the tools to be able to interpret what he hears, sees, and feels in how he communicates with people. Once a person is aware of the thought process, they will have the tools to change their words and behaviors to achieve what they want. Mental program is one of the topics discussed in the NLP workshop. For example, the subjects were taught how to use appropriate language patterns to influence the other person according to their mental programs. Appropriate language allows the subjects to convey their messages correctly to the other person and become effortlessly intimate with them. Therefore, it can be said that increasing learners' EI can be due to teaching NLP strategies such as good comprehension, intimacy, sensory awareness, thinking about the outcome, and behavioral flexibility.

## Conclusion

Neurolinguistic programming strategies play an effective role in learners' critical thinking, academic achievement, and EI in EFL Learners' advanced level. This research addressed the following hypotheses:

1. NLP strategies play an influential role in EFL learners' critical thinking in advanced language proficiency.
2. NLP strategies influence/contribute to learners' academic achievement in EFL Learners' advanced language proficiency.
3. NLP strategies influence the EFL learners' EI in advanced language proficiency. It is suggested that other NLP techniques and patterns need to be considered. It is also suggested that different educational levels should be studied in similar future studies. In addition, it is suggested that educators make it possible for parents and student teachers to use such teachings technique. In the shadow of the application of these teachings technique, we can see the improvement of the quality of teachers' teaching.

## Suggestions

1. While this study focused on teaching NLP, critical thinking, academic achievement, and emotional intelligence, it is suggested to take other skills into account in other studies.
2. The participants of this study were EFL at the advanced level; other studies can be conducted to study the instruction of NLP to different levels.
3. The participants of this study were women; another study can be conducted to study the impact of NLP on men, or the combination of both male and female learners.
4. Since the main objective of strategy instruction is to equip learners with the ability to use them on their own, it seems reasonable that other research studies investigate the impact of NLP on developing EFL learners' autonomy.

## Limitations

Similar to other studies, this study had limitations on sample size, the field of study, and gender, and it is suggested that future studies of both genders and other groups be considered in educational settings.

## Implication

The findings of this study can offer pedagogical implications for different practitioners in the field.

### Implications for teachers

At present, language teaching is intermingled with the emphasis on giving more prominence to cognitive strategies and the quality of thinking. Furduescu (2019) believes enhancing students' critical thinking abilities is the core of meaningful education. To meet these requirements in the classroom, there is no way but to modify the routines of instruction. Ghaemi and Taherian (2011) asserted that “the time of manual teachers, those teachers who just follow the teaching instruction, is over. The educational system needs teachers who are researchers and in line with the new methods in teaching” (p. 9).

### Implications for EFL learners

At present, language teaching process requires participation of both teachers and learners. Therefore, the results of the current study have implications for language learners as well; encouraging them to become more analytic in thinking, using critical thinking skills, enhancing academic achievement, and emotional intelligence.

Furthermore, improving NLP strategies in a learning context can help learners to be equipped with strategies designed to have positive attitudes toward learning and positive views of themselves as learners. When learners are empowered with NLP strategies in tandem with learning strategies, they will have more awareness of the process in their mind during learning.

### Implications for EFL syllabus designers, curriculum developers, and teacher educators

Having a significant role in language learning settings, syllabus designers can infuse strategies of NLP, critical thinking, and their combination into the materials in coursebooks with the aim of empowering students more and more by means of these techniques and strategies.

Having strategies of critical thinking, NLP, and their combination in a systematic way in reading comprehension and vocabulary retention tasks will provide learners with a suitable opportunity to learn and use them and consequently foster their deep understanding of the written text, and grasp potential implications within the text and read between the lines. Form the aspect of vocabulary retention, these strategies may lead to challenge the source of knowledge in the lexicon inside their mind. Also, teacher educators and teacher trainers need to first make teachers aware of the nature and benefits of these strategies, and second train teachers to implement them in the classes. EFL practitioners should arrange some in-service courses for EFL teachers to familiarize them with these strategies and their benefits and advantages in teaching.

## Data availability statement

The original contributions presented in the study are included in the article/supplementary material, further inquiries can be directed to the corresponding authors.

## Author contributions

All authors listed have made a substantial, direct, and intellectual contribution to the work and approved it for publication.
